# Current Hospital Topics

**Published:** 1906-07-28

**Authors:** 


### HOSPITAL ADMINISTRATION.

### CONSTRUCTION AND ECONOMICS.

### CURRENT HOSPITAL TOPICS.

### LESSONS FROM AUSTRALASIA.

#### Election and Service of Honorary Medical Staff.

From time to time the mode of election and terms of appointment of honorary medical officers to the hospital staff comes up for discussion. The best system is undoubtedly that which provides for the appointment of an election committee each year, consisting of one hundred governors, in which number are included the members of the committee of management for the time being, certain honorary officials, and a number of independent governors selected by ballot from the whole body, so as to increase the committee to one hundred. It is now the practice at the best administered hospitals in this country to provide in the rules that honorary medical officers shall retire at 60, and that they shall not be eligible for re-election. At a special general meeting of the Melbourne Women's Hospital a discussion recently took place on these questions, and it was decided to vest the election of the honorary medical staff, twelve in number, in the hands of the committee, each honorary medical officer being elected for a period of seven years; on attaining the age of 60 years honorary medical officers to cease to hold office in the hospital. It was agreed that the new regulations should not lengthen or shorten the terms of office of any honorary medical officer appointed before the byelaw came into operation. These alterations, though strenuously opposed, were adopted by a large majority. We feel the governors of the Melbourne Women's Hospital would be well advised to vest the election of the honorary medical officers in an election committee of one hundred governors on the plan we have already explained.

#### Royal Prince Alfred Hospital, Sydney.

The importance attaching to the visit of inspection which the secretary, Mr. William Epps, has made to American and European hospitals is shown in a practical manner by the proceedings of a special meeting of the board of governors recently held. It was resolved, on the recommendation of the secretary, to establish a Hospital Sunday Fund, a Ladies' Auxiliary Society to make and provide linen, a Hospital Alliance of young people to undertake special duties, to extend the area in which collections for the hospital are made, to reorganise the system of receiving casualty patients, and to register the names and diseases of all in and out patients on the card system. It was further determined to exact a payment from out-patients, except in extreme cases of destitution, of (1) 3d. for each supply of medicine; (2) 3d. for each new bottle supplied; (3) 2d. for each surgical dressing. The laundry was to be reorganised and new machinery purchased, whilst a new and thorough system was introduced to check the issue and receipt of all linen through the workroom organisation. The ward sisters' salaries were increased to £80 a year, and first-year payments to probationers were abolished. A system of internal telephones is to be introduced; certain of the ward floors are to be treated with a petroleum preparation, and a plant to manufacture aerated waters for the hospital was ordered. A committee consisting of Professor Anderson Stuart, Sir James Graham, Sir James Fairfax, and Mr. C. K. Mackellar was appointed to consider the advisability of (1) affiliating with the Royal National Pension Fund for Nurses; (2) establishing forthwith a preliminary training school for nurses, and a nursing hostel, or the institution of a private nursing service; (3) the question of purchasing meat in carcase and some other stores in the open market instead of by contract; and (4) the adoption of asbestolith for covering the old ward floors. The same committee has under consideration a system of cold storage for the mortuary, with ice-making plant, and the adoption of the Paul system of vacuum steam heating. It would be impossible to better illustrate the practical utility of arranging for the chief official of the larger hospitals to make himself acquainted with the methods pursued in other countries in the management of their hospitals.

#### A Salvation Army Hospital.

The Salvation Army has opened the first hospital it has ever established, at Melbourne, to be known as the Bethesda Hospital. The building contains thirteen wards, and has accommodation for 52 beds, of which 42 are provided as a commencement. The institution is designed to supply a link between the public hospitals and the private hospitals, where high fees are charged. The hospital is to be self-supporting, and all classes will be eligible for admission on payment. There are three classes of accommodation, that for the rich, that for patients who can pay a remunerative rate, and that for those who can only provide out-of-pocket expenses. The nursing will be done by Salvation Army sisters who have been properly trained for the work, and there is to be a complete system of district nursing in connection with the hospital. The building was formerly occupied as a spacious private residence, and £2,000 has been spent upon its conversion to hospital purposes. This experiment will be watched with interest everywhere, for it aims at supplying a want which is admitted to be felt in regard to hospital accommodation in the old country at the present time.

#### State and Rate Support.

In the Colonies the hospitals are largely or entirely supported by the Government out of rates and taxes. It has always been held, and rightly held, that such contributions cannot be made to public institutions without involving representation and the practical control of the management. In New Zealand, for instance, the local bodies who contribute to the support of the hospitals elect the members of the board of management. This system appears to have given rise to some criticism, and at a recent meeting of the Hospital and Charitable Aid Board a resolution was proposed to recommend to the Government that all members of hospital and charitable aid boards should in future be elected by the ratepayers. This resolution was defeated, but it indicates the trend of public opinion and shows one inevitable consequence of giving up voluntary support in favour of Government grants and rate aid. It appears from the discussion which took place at the meeting referred to that directly the Government extends financial aid to a hospital the voluntary contributions gradually fall away, and it becomes more and more difficult to secure gifts of money for such hospitals from the public. Fortunately there is no probability that the voluntary hospitals in Great Britain need ever accept State aid so long as the management is vigorous and efficient. We proved this by the publication in this column a few weeks ago of figures which exhibited the present position of the London hospitals.

